# Individual Variability in Deep Learning-Based Joint Angle Estimation from a Single IMU: A Cross-Population Study

**DOI:** 10.3390/s26010178

**Published:** 2025-12-26

**Authors:** Koyo Toyoshima, Jae Hoon Lee, Shigeru Kogami, Teppei Miyaki, Toru Manabe

**Affiliations:** 1 Graduate School of Science and Engineering, Ehime University, Bunkyo-cho 3, Matsuyama 790-8577, Ehime, Japan; m801032b@mails.cc.ehime-u.ac.jp; 2 Graduate School of Medicine, Ehime University, Shitsukawa, Toon 791-0295, Ehime, Japan; kogami.shigeru.xj@ehime-u.ac.jp; 3 Rehabilitation Center, Ehime University Hospital, Shitsukawa, Toon 791-0295, Ehime, Japan; miyaki.teppei.mf@ehime-u.ac.jp (T.M.); manabe.toru.pe@ehime-u.ac.jp (T.M.)

**Keywords:** gait analysis, inertial measurement unit, wearable sensors, deep learning, joint angle estimation, older adults, hip osteoarthritis, generalization, individual variability

## Abstract

Walking ability is crucial for maintaining independence and healthy aging. Although joint angle measurement is important for detailed gait assessment, it is rarely performed in clinical practice due to the complexity of motion capture systems. This study investigates individual variability and cross-population generalizability of deep learning-based joint angle estimation from a single inertial measurement unit (IMU) attached to the pelvis. Gait data from three distinct populations were collected: 17 young adults, 20 healthy older adults (aged 65+), and 14 pre-operative patients scheduled for hip replacement surgery due to hip osteoarthritis (also aged 65+). A 1D ResNet-based convolutional neural network was trained to estimate bilateral hip, knee, and ankle joint angles from IMU signals. We systematically compared within-population training (trained and tested on the same population) with cross-population training (trained on combined data from all populations) using nested 5-fold cross-validation. Cross-population training showed population-specific effectiveness: older adults demonstrated consistent improvement, while young adults showed minimal change due to already high baseline performance, and pre-operative patients exhibited highly variable responses. These findings suggest that the effectiveness of cross-population learning depends on within-population gait heterogeneity, with important implications for developing clinically applicable gait analysis systems across diverse patient populations.

## 1. Introduction

Walking ability is fundamental to maintaining independence and quality of life, particularly in the older adult population. The significance of walking as a vital functional activity is widely recognized in healthcare settings, as it reflects multiple aspects of an individual’s health status, including motor control, muscle performance, sensory function, endurance, cognitive status and overall well-being [1]. In clinical practice, the assessment of gait parameters serves as a crucial tool for identifying mobility impairments, fall risk [2], frailty [3], Parkinson’s disease [4] and the progression of various age-related conditions. Given its critical role in health assessment, gait analysis is widely implemented across various healthcare settings, including rehabilitation centers, orthopedic clinics, and research institutions. It serves as an essential tool for treatment planning and progress evaluation [5].

Gait analysis methods vary depending on the setting. In research institutions, detailed biomechanical data can be obtained using optical motion capture systems and force plates. However, these methods require larger space, specialized facilities and expertise, and are time-consuming, making them impractical for routine clinical use. In contrast, clinical assessments are primarily conducted through visual observation by physicians and physical therapists, along with standardized tests such as Timed Up and Go (TUG) test [6]. The TUG test measures the time required for an individual to stand up from a chair, walk three meters, turn around, walk back, and sit down again.

These clinical assessments are practical and widely used due to their ease of implementation in daily practice. However, they may lack the objective and quantitative data necessary for detailed gait analysis. Indeed, visual gait assessment has limitations in reliability, with reports indicating that only a portion of predicted gait deviations can be detected and that inter-rater and intra-rater agreement remains at moderate levels [7,8]. Furthermore, Barry et al. reported that the TUG test has limited ability to predict falls in community-dwelling older adults and should not be used in isolation [9]. While instrumented gait analysis (IGA) has been available for over 30 years and has demonstrated potential to improve clinical outcomes, its use remains largely confined to research institutions due to practical barriers including high costs, complex equipment, and the need for specialized expertise [10,11].

Among various gait parameters, including spatiotemporal measures such as walking speed and step length, joint kinematics plays a particularly crucial role in comprehensive gait analysis. While spatiotemporal parameters provide important information about overall gait characteristics, joint angle measurements offer more detailed insights into movement patterns. They provide quantitative and reproducible assessment that enables clinicians to identify subtle abnormalities that may not be apparent through visual observation or simple spatiotemporal measures alone.

The reliability of kinematic data has been well established. Kadaba et al. demonstrated excellent repeatability of joint angles in the sagittal plane both within and between test sessions, suggesting that clinical decisions can reasonably be based on a single gait evaluation [12]. Beyond basic gait assessment, joint angle measurements serve practical clinical purposes. For instance, Alrawashdeh et al. demonstrated that joint angle symmetry measured by inertial sensors can be used for pre- and post-operative planning in total knee arthroplasty, providing objective targets for surgical intervention and rehabilitation [13]. Furthermore, joint kinematics enables comprehensive gait assessment through composite indices such as the Gait Deviation Index, which integrates multiple kinematic features into a single score representing overall gait pathology [14]. These findings underscore that detailed joint angle measurements are fundamental for both clinical assessment and treatment planning.

Recent advances in sensing technologies and machine learning, especially deep learning, have enabled practical gait analysis outside laboratory settings. Various approaches have been developed using monocular cameras [15,16], range sensors including LiDAR and millimeter-wave radar [17,18], and insole pressure sensors [19]. These methods have shown promising results in quantifying gait parameters in diverse environments.

Among these technologies, IMU sensors are particularly promising for clinical gait analysis due to their compact size, low cost, and ease of use. These sensors typically integrate accelerometers, gyroscopes, and sometimes magnetometers to measure linear acceleration, angular velocity, and magnetic field orientation, respectively. Various approaches have been developed for IMU-based gait analysis. Many studies have focused on extracting spatiotemporal parameters such as walking speed, step length, cadence, and gait phases from sensor signals [20]. These approaches have demonstrated reliable estimation of basic gait parameters using single or multiple IMU sensors placed on specific body segments, with applications extending to pathological populations including elderly adults and patients with Parkinson’s disease [21].

More recently, advances in deep learning-based pose estimation have enabled a more comprehensive analysis by reconstructing full-body kinematics from a minimal number of IMU sensors. Wang et al. employed a hybrid temporal convolutional neural network-bidirectional LSTM model using three IMUs to estimate hip, knee, and ankle joint angles and moments during various locomotive activities [22]. Lim et al. demonstrated that lower limb kinematics during walking can be estimated from a single IMU placed on the lower back using machine learning, supporting the feasibility of inferring distal joint motion from pelvic measurements [23]. Alemayoh et al. showed that a single IMU attached to various lower-body segments could estimate leg joint angles during walking with mean absolute errors around 2–4° [24,25].

The feasibility of estimating full lower-limb kinematics from a single point on the trunk is supported not only by empirical results but also by biomechanical theory. From a mechanical perspective, gait is governed by physical constraints that link the motion of the center of mass (CoM) to the distal joints. Lim and Park demonstrated that an extended spring-loaded inverted pendulum model with a compliant, off-centered curvy foot can emulate lower-limb kinematics, including ankle joint kinematics and associated joint torque, across various walking speeds [26]. This suggests that the seemingly complex multi-joint coordination of gait exhibits structured, low-dimensional patterns determined by passive dynamics. Such physical coupling between the pelvis (approximating the CoM) and the lower limbs provides a theoretical justification for using data-driven models to infer joint angles from a single pelvic sensor.

However, most existing studies have primarily focused on healthy young adults. Relatively few have systematically investigated the performance of deep learning-based joint angle estimation across diverse populations including older adults and patients with pathological gait patterns. The generalizability of deep learning models across different population groups remains an important open question. This is particularly relevant given the known differences in gait characteristics between young and older adults [27] and the high variability observed in pathological conditions such as hip osteoarthritis [28].

This study investigates the individual variability and cross-population generalizability of deep learning-based joint angle estimation from a single IMU sensor attached to the pelvis. Gait data were collected from three distinct populations: healthy young adults, older adults, and pre-operative patients scheduled for hip replacement surgery. Using a 1D convolutional neural network, within-population training (models trained and tested on the same population) was systematically compared with cross-population training (models trained on combined data from all populations). This work makes three contributions: (1) a systematic comparison of within-population and cross-population training across three distinct populations with different gait characteristics, (2) a detailed analysis of individual-level variability in response to cross-population training within each population, and (3) insights into population-specific differences in cross-population training effectiveness across the three studied populations.

## 2. Methods

### 2.1. Dataset

#### 2.1.1. Participants

Three distinct groups were recruited for this study: young adults, older adults, and pre-operative patients scheduled for hip replacement surgery. The young adult group (*n* = 17) had no history of neurological or orthopedic conditions. The older adult group (*n* = 20) comprised individuals aged 65 years and older who were capable of independent walking without assistive devices. The pre-operative patient group (*n* = 14) consisted of individuals scheduled to undergo hip replacement surgery the following day, representing individuals with pathological gait patterns due to hip osteoarthritis (OA). Although all patients were capable of independent walking without assistive devices, they exhibited pathological gait patterns characterized by reduced walking speed and balance impairments caused by joint pain and cartilage deformation. The severity varied within the group. Many participants demonstrated limited endurance (e.g., walking only 3–4 round trips), while others walked similarly to healthy individuals due to relatively mild symptoms. Detailed demographic information is provided in Table 1.

All personal data were anonymized using identification numbers (Y01–Y17 for young adults, O01–O20 for older adults, and P01–P14 for pre-operative patients). All participants gave their informed consent for inclusion before participating in this study. This study was conducted in accordance with the Declaration of Helsinki and was approved by the Ethics Committee of Ehime University (Project identification code: 2303002) on 27 March 2023.

#### 2.1.2. Measurement System

**Figure 1:**
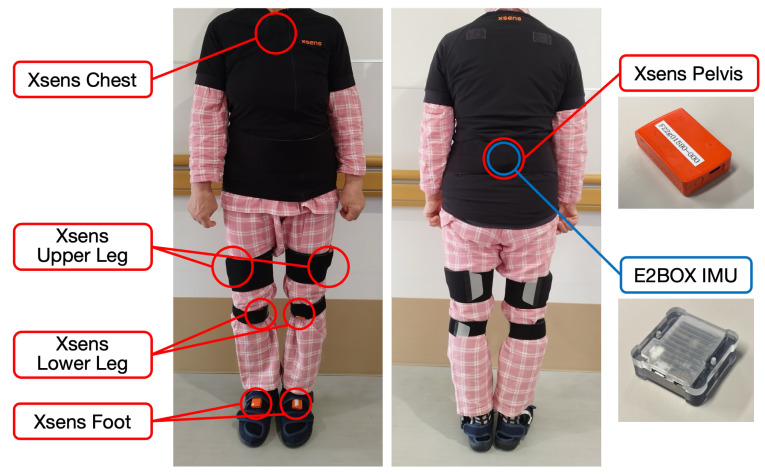
Sensor placement configuration. The blue sensor represents the E2BOX IMU attached to the pelvis, and the red sensors represent the eight Xsens motion capture sensors.

During data collection, participants were equipped with an IMU sensor (AHRS EBIMU-9DOFV5-R3, E2BOX Co., Ltd., Hanam, Republic of Korea, hereafter referred to as “E2BOX IMU”) attached to their pelvis. The E2BOX IMU was configured in local linear acceleration mode with gravity removal enabled, and raw sensor data (3-axis accelerometer and 3-axis gyroscope) were served as the input to the models. Simultaneously, an IMU-based motion capture system (Xsens MTw Awinda, Xsens Technologies B.V., Enschede, The Netherlands, hereafter referred to as “Xsens”) with 8 sensors was used to record their joint kinematics as ground truth data. The Xsens system was configured with the “Lower Body with Sternum” body model, which uses sensors placed on the pelvis, sternum, both thighs, both shanks, and both feet. Both systems collected data at a sampling frequency of 100 Hz. Figure 1 shows the experimental setup with sensor placements on a participant.

#### 2.1.3. Data Collection Protocol

For data collection, participants were instructed to walk along an 18-m flat, hard-surface straight path indoors. The walking protocol differed between populations: young adults and older adults were asked to walk for up to 10 min, with the protocol divided into three equal periods during which they walked at three different self-selected speeds (normal, slow, and fast). This protocol was designed to capture a comprehensive dataset of walking patterns across different speeds. In contrast, pre-operative patients walked at a single self-selected comfortable speed due to their physical limitations, and many participants, particularly in the pre-operative group, walked for less than 10 min. Throughout the experiment, all participants could stop at any time if they felt any physical discomfort. To synchronize E2BOX IMU and Xsens after collecting data, the pelvis sensor was tapped at the beginning and end of the data collection. This provided clear acceleration peaks for synchronization.

#### 2.1.4. Data Preprocessing

Due to occasional data loss in the E2BOX IMU recordings, data segments with consecutive missing values exceeding 5 frames (50 ms at 100 Hz) were excluded. For gaps of 5 frames or less, linear interpolation was applied to reconstruct the missing data. Following this interpolation, the E2BOX IMU and Xsens data were synchronized using acceleration peaks from sensor tapping at the beginning and end of the data collection.

#### 2.1.5. Dataset Creation

The dataset was created using a sliding window approach. A fixed-size window of 200 samples (2 s) was sequentially shifted forward with a stride of 10 samples (100 ms), resulting in a 95% overlap between consecutive windows. Each window contained 6-channel IMU data (3-axis accelerometer and 3-axis gyroscope) as input, with the corresponding six joint angles (bilateral hip, knee, and ankle flexion/extension) at the final time step as the output target.

**Figure 2:**
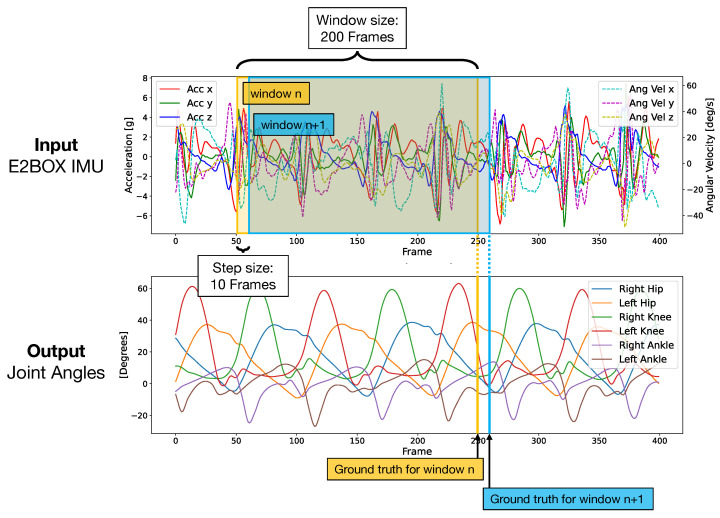
Dataset creation process using a sliding window approach. Sequential IMU data (6-channel: 3-axis accelerometer and 3-axis gyroscope) was divided into overlapping windows of 200 samples (2 s) with a stride of 10 samples (100 ms). Each window serves as input to estimate the corresponding six joint angles at the final time step.

This approach, which uses only past and current sensor data to estimate the current joint angles, was designed to maintain real-time applicability. The dataset was split on a subject-wise basis for cross-validation. Figure 2 illustrates this dataset creation process.

### 2.2. Deep Learning Model

In this study, we employed a 1-Dimensional Convolutional Neural Network (1D CNN) to estimate lower extremity joint angles during walking. The 1D CNN architecture excels in processing temporal sequences by applying convolution operation along a single time axis, making it particularly suitable for analyzing sequential gait data. Compared to other deep learning approaches such as Recurrent Neural Network (RNN), 1D CNNs provide a simpler architecture while maintaining high performance in time-series analysis. 1D CNNs have demonstrated effectiveness in various applications involving 1D signals, such as biomedical data classification, structural health monitoring, and anomaly detection, offering advantages in real-time processing and efficient hardware implementation [29]. We selected 1D CNN for this study considering its potential for real-time implementation in future applications.

The proposed 1D CNN model follows a ResNet-based architecture that processes 6-channel IMU signals (3-axis accelerometer and 3-axis gyroscope) with a 200-frame window. The network begins with a convolutional stem containing a convolution and max pooling layer for initial feature extraction. This is followed by four residual stages with progressive channel dimensions (64 → 128 → 256 → 512). Downsampling is performed using a stride of 2 in the first block of the last three stages. Each residual stage contains BasicBlocks with skip connections to facilitate gradient flow. After global average pooling, the features are passed to a regression head composed of three linear layers each followed by GELU and dropout (*p* = 0.2), followed by a final linear output layer. The output comprises 6 joint angles representing bilateral hip, knee, and ankle flexion/extension movements.

**Figure 3:**
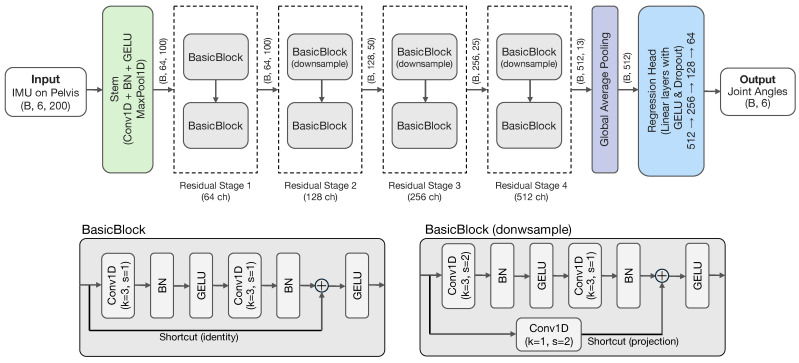
Network architecture of the 1D ResNet-based model for joint angle estimation from IMU signals.

The total number of model parameters is 4,019,014 (FP32 model size: 15.33 MB). Figure 3 illustrates the overall architecture of the proposed model, and Table 2 provides the detailed layer specifications.

### 2.3. Training and Validation Strategy

Prior to training, all participants were randomly assigned to five folds for cross-validation. The fold assignment was fixed to ensure reproducibility across all experiments (see Appendix A for detailed participant assignment). All input features and output targets were standardized using z-score normalization, where the mean and standard deviation were computed from the training data only and then applied to validation and test data to prevent data leakage.

Based on these fixed assignments, we implemented two distinct training settings.

First, the within-population strategy served as a baseline, where training and testing were performed within each group. Second, the cross-population strategy was designed as a global modeling approach, aiming to learn a generalized representation from all available populations. In this setting, participants with the same fold number across all groups were combined. For each validation iteration (e.g., testing on Fold *k*), a new model instance was initialized and trained on the combined data from the remaining folds (Folds ≠k) of all three populations to ensure strict participant independence.

**Figure 4:**
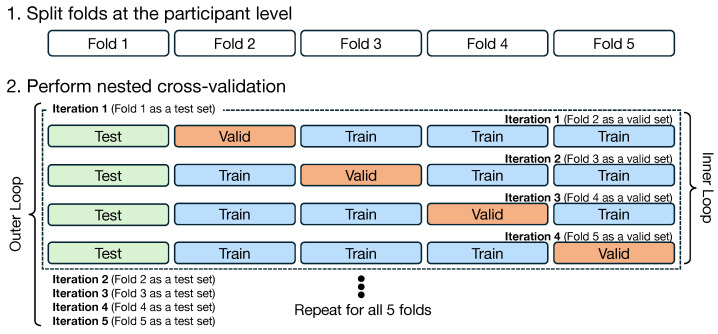
Training strategy: (1) participants were split into five folds at the participant level, and (2) nested cross-validation was performed with an outer loop for testing and an inner loop for training and validation. Colors indicate the data split: training (blue), validation (orange), and test (green).

Model training was performed using a nested cross-validation approach. In the outer loop, each of the five folds was sequentially used as the test set. In the inner loop, the remaining four folds were further split using 4-fold cross-validation: one fold served as the validation set and the other three folds as the training set. This process was repeated four times with each fold serving as the validation set once, and the validation results were averaged. Figure 4 illustrates this training strategy, showing both the participant-level fold splitting and the nested cross-validation process.

To address the imbalance in the number of data samples across participants due to different walking durations, weighted random sampling was applied during training. Each participant’s sampling weight was calculated as the inverse of their number of sliding windows, ensuring balanced representation of all participants regardless of their walking duration. This weighted sampling was applied only to the training data, while validation and test data were processed sequentially without sampling.

### 2.4. Implementation Details and Real-Time Feasibility

Hyperparameters were determined empirically through preliminary experiments and kept fixed across all folds to ensure fair comparison. For each fold, the model was trained for 100 epochs with a batch size of 128, using the Adam optimizer with a learning rate of 0.001 and Huber loss function. All experiments were conducted on an NVIDIA GeForce RTX 3090 GPU.

To assess the feasibility of real-time application, we analyzed the computational efficiency of the model on this hardware. We measured the inference latency for a single input window (batch size = 1) averaged over 1000 iterations. The mean inference time was 2.83 ms on the CPU and 2.41 ms on the GPU. These processing times are sufficiently short, confirming the model’s capability for real-time processing.

### 2.5. Statistical Analysis

Model performance was evaluated using Mean Absolute Error (MAE) and Pearson correlation coefficient (R). We defined ΔMAE = MAE_Cross_− MAE_Within_ (negative values indicate improvement) and ΔR = R_Cross_− R_Within_ (positive values indicate improvement). Statistical significance was tested using a two-sided Wilcoxon signed-rank test for each population and each metric. For the mean Δ values, 95% confidence intervals were computed using a percentile bootstrap with 10,000 resamples. To account for multiple comparisons, Bonferroni correction was applied across 6 tests (3 groups × 2 metrics). Effect size was reported as $r=z/\sqrt{n}$, where *z* is the standardized normal deviate from the Wilcoxon signed-rank test and *n* is the number of paired observations used in the test (i.e., excluding zero differences). The sign of *r* was determined by the median of the paired differences (cross-population−within-population).

## 3. Results

### 3.1. Population-Level Performance

**Figure 5:**
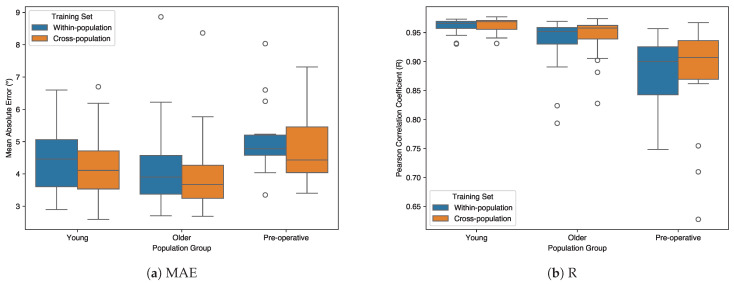
Performance comparison between within-population and cross-population training. (**a**) Boxplot of Mean Absolute Error (MAE). (**b**) Boxplot of Pearson correlation coefficient (R). Open circles indicate outliers (beyond 1.5 × IQR).

Figure 5 presents the distribution of estimation errors for within-population and cross-population training strategies across all three populations. Each boxplot shows the distribution of average MAE or R values across all six joints (bilateral hip, knee, and ankle) for each participant. The box represents the interquartile range (IQR, 25th to 75th percentile), the horizontal line within the box indicates the median, whiskers extend to 1.5 × IQR, and open circles indicate outliers beyond this range.

For MAE (Figure 5a), all three populations showed median reductions with cross-population training. The young adult group showed a median reduction of 0.35° with decreased interquartile range (IQR: 1.46° to 1.18°) and introduced one outlier. The older adult group demonstrated a median reduction of 0.23° with decreased IQR (1.20° to 1.02°) and maintained one persistent outlier. The pre-operative group showed a median reduction of 0.35° but with substantially increased IQR (0.63° to 1.41°); notably, the four outliers present in within-population training were eliminated in cross-population training. The pre-operative group exhibited the highest median MAE values and widest ranges in both training conditions.

For correlation coefficients (Figure 5b), the young adult group maintained consistently high median R values (0.966 to 0.969) with decreased number of outliers (three to one). The older adult group showed an increase in median R from 0.952 to 0.958, though the number of outliers slightly increased from two to three. The pre-operative patient group showed the lowest median R values (0.900 to 0.907) and widest IQR (0.083 to 0.067), with the number of outliers increasing from zero to three with cross-population training.

While the boxplots above show the median values and their distributions, Table 3 and Table 4 provide detailed joint-specific mean performance across all participants within each population. For MAE (Table 3), cross-population training reduced errors across all joints in all populations, with the most substantial improvements observed in the knee joint for older adults ($\Delta =-0.47^{\circ }$) and the hip joint for young adults ($\Delta =-0.42^{\circ }$). For correlation coefficients (Table 4), the changes were minimal across all populations, with young adults and older adults showing slight improvements (maximum Δ=+0.013 for older adults’ knee joint). Pre-operative patients showed slight decreases in hip and knee correlations with cross-population training.

### 3.2. Statistical Comparison

Table 5 and Table 6 present the statistical comparison between within-population and cross-population training. In young adults, MAE showed no statistically significant difference. *R* showed a small increase in the uncorrected analysis, but this effect did not remain statistically significant after multiple-comparison correction. In older adults, cross-population training showed statistically significant differences for both MAE and *R*, and these results remained significant after Bonferroni correction. The corresponding effect sizes were large, and the 95% confidence intervals for the mean differences excluded zero. In pre-operative patients, neither metric showed a statistically significant group-level difference, and the 95% confidence intervals included zero.

### 3.3. Individual Variability

The individual responses to cross-population training varied considerably both within and across populations, reflecting the complex interplay between population characteristics and individual gait patterns.

Table 7 presents representative participants from each group showing the range of individual responses to cross-population training, with complete results for all participants provided in Appendix B.

In the young adult group, 10 out of 17 participants (59%) showed reduced MAE with cross-population training, though the magnitude of improvement was generally modest. The largest improvement was observed in participant Y10 (ΔMAE = $-1.94^{\circ }$), while the largest degradation occurred in Y12 (ΔMAE = $+0.64^{\circ }$). Despite these individual variations, most participants maintained high correlation coefficients (R>0.93) regardless of training strategy. The older adult group demonstrated the most consistent pattern of improvement: 16 out of 20 participants (80%) showed reduced MAE, and 12 participants (60%) showed improved R values. Notable improvements were observed in participants O08 (ΔMAE = $-0.90^{\circ }$, ΔR = +0.058), O12 (ΔMAE = $-0.50^{\circ }$, ΔR = +0.042), and O17 (ΔMAE = $-0.74^{\circ }$, ΔR = +0.014). These participants had relatively high baseline errors and lower correlation coefficients under within-population training. Only 4 participants showed slight increases in MAE, with the largest being O11 (ΔMAE = $+0.30^{\circ }$). The pre-operative patient group exhibited the highest variability in individual responses. Seven participants showed improved MAE, while the other seven showed degradation. Participant P11 demonstrated the largest improvement (ΔMAE = $-1.55^{\circ }$, ΔR = +0.062), whereas participant P12 showed substantial degradation (ΔMAE = $+1.06^{\circ }$, ΔR = −0.193). Similarly, for correlation coefficients, 7 participants improved and 7 worsened.

### 3.4. Visualization of Representative Cases

To illustrate the population-specific effects of cross-population training, we selected representative participants from each group based on objective criteria. For each population, participants were identified whose within-population MAE was closest to the group median. From the young adult group, participant Y05 was selected (MAE: $4.46^{\circ }$, group median: $4.46^{\circ }$). From the older adult group, participant O09 was chosen (MAE: $3.97^{\circ }$, group median: $3.91^{\circ }$). For the pre-operative patient group, two participants were selected to demonstrate the heterogeneous responses observed in this population. Participants were divided into two subgroups based on whether cross-population training improved or worsened their performance (ΔMAE <0 or ΔMAE >0). From each subgroup, the participant closest to the subgroup median was chosen: P01 (MAE: $4.74^{\circ }$, subgroup median: $4.74^{\circ }$) from the improved subgroup (n=7), and P08 (MAE: $4.82^{\circ }$, subgroup median: $4.82^{\circ }$) from the degraded subgroup (n=7).

**Figure 6:**
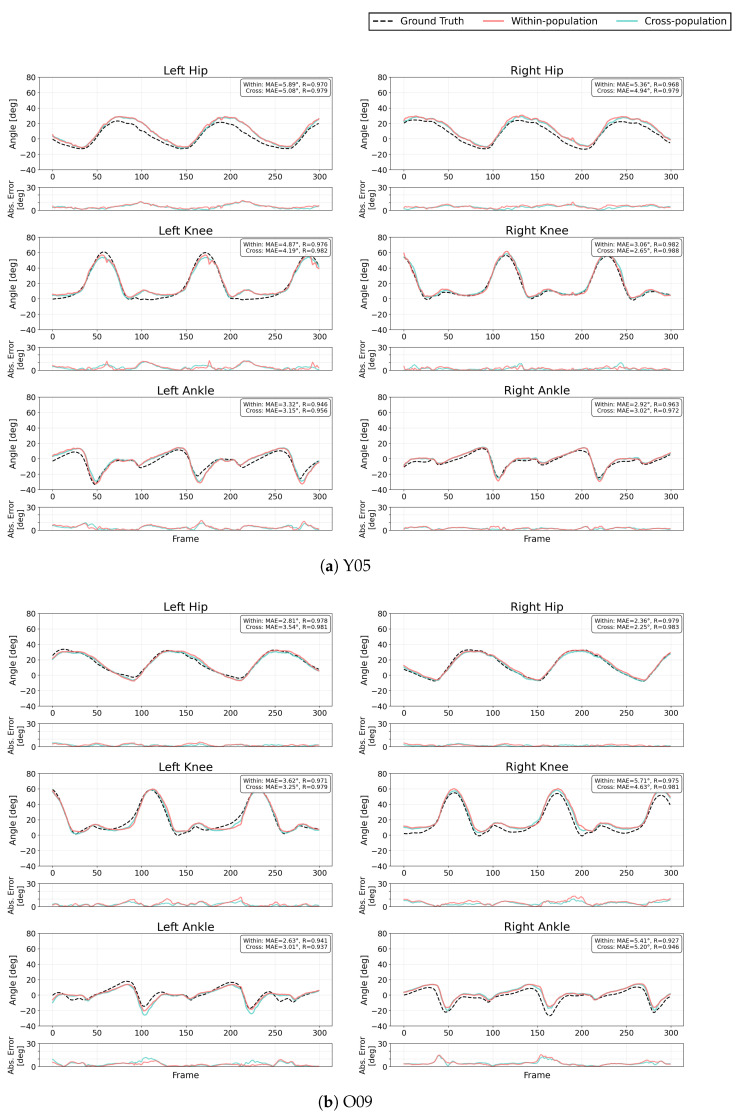

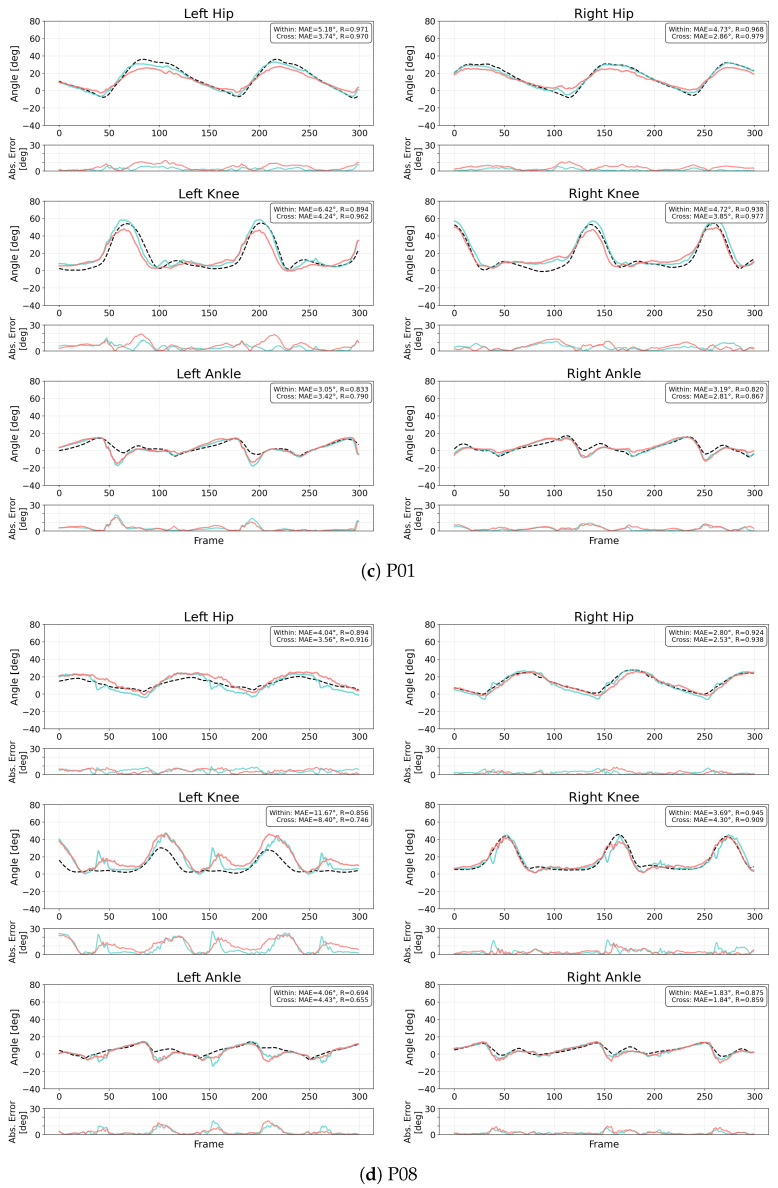
Representative joint angle estimation examples for selected participants. Black dotted lines show ground truth from Xsens motion capture system. Blue lines represent cross-population training estimations and red lines represent within-population training estimations. For each joint and side, the upper panels show the estimated joint angles, the lower panels show the absolute estimation error (|estimated angle − ground truth|). Each estimation curve represents the mean output of the four inner-fold models within the nested cross-validation. (**a**) Y05, (**b**) O09, (**c**) P01, and (**d**) P08.

Figure 6 presents time-series joint angle estimations for these representative participants, comparing ground truth measurements with estimations from both training strategies. For each participant, bilateral hip, knee, and ankle joint angles are displayed during continuous walking. Each estimation line shows the mean of four inner-fold models from the nested cross-validation framework.

## 4. Discussion

This study examined the effectiveness of cross-population training for IMU-based joint angle estimation across three distinct populations. Our findings suggest that the benefits of cross-population training vary substantially depending on the inherent gait heterogeneity within populations. Cross-population training showed different effects across the three populations studied. Older adults showed consistent improvement with statistically significant group-level reduction in estimation error. In contrast, young adults showed minimal change, and pre-operative patients showed highly variable individual responses. These divergent patterns suggest that the effectiveness of cross-population training depends on both population-level characteristics and individual gait variability.

In interpreting these results, it is important to note that improvements in MAE and R alone should not be equated with clinical significance. Although MAE provides a useful quantitative measure for comparing model performance, clinically acceptable error ranges for joint angle estimation have not been uniformly established. They may differ depending on the clinical context and intended application. Accordingly, the MAE and R differences observed here are discussed in terms of model generalization across populations and individuals, rather than as indicators of direct clinical benefit.

Young adults showed minimal improvement from cross-population training. This finding is not surprising given that their baseline performance was already high (mean R = 0.96). When individuals within a population walk similarly to each other and within-population models already perform this well, there is little room for further improvement. This suggests that for young, healthy individuals, individual-specific models may be sufficient, and cross-population approaches offer limited practical benefit.

In contrast, older adults showed consistent improvement, with the majority of participants showing reduced MAE and improved R. This finding aligns with evidence that the older adult population exhibits greater within-group heterogeneity in gait patterns [27,30]. When individuals within a population show diverse movement patterns, a model trained only on similar individuals may struggle to capture this full range of variability. Cross-population training may help by exposing the model to more diverse movement patterns. Training on young adults (who walk with larger, faster movements) and pre-operative patients (who walk with slower, more cautious patterns) may provide the diversity needed to better represent the heterogeneous older adult population.

Pre-operative hip replacement patients showed highly variable individual responses to cross-population training. This heterogeneity is consistent with previous findings that hip OA patients exhibit high gait variability due to fluctuating pain intensity, muscle fatigue, and unstable movement patterns [28]. This high within-group variability makes this patient population particularly challenging for cross-population training. Statistically, this heterogeneity was reflected in the wide 95% confidence intervals ([−0.53, +0.19] for MAE), which span across zero. Some patients improved substantially (e.g., P11: ΔMAE = $-1.55^{\circ }$, ΔR = +0.062), while others showed marked degradation (e.g., P12: ΔMAE = $+1.06^{\circ }$, ΔR = −0.193). This may reflect those with severe pain-related compensations or asymmetric gait patterns not represented in the training data from healthy young and older adults.

The unpredictable nature of results in this population highlights an important clinical consideration. Unlike older adults where cross-population models can be applied more reliably, pre-operative hip replacement patients require individual validation to ensure model accuracy before clinical use.

Several limitations should be acknowledged. First, this study focused on straight-line walking, which represents the most fundamental and clinically relevant gait pattern for rehabilitation assessment. While this focus is appropriate for many applications in gait analysis and clinical evaluation, the applicability of our single pelvis-mounted IMU approach to more complex movements (e.g., stair climbing, turning, or running) remains to be explored. Such complex movements may require additional sensors or different sensor configurations.

Second, we did not examine which gait characteristics (e.g., walking speed, step length, or movement variability) were associated with successful cross-population training. Future studies should identify predictors of cross-population model performance.

Third, the sample size was relatively small and the demographics were not fully balanced, in particular for the pre-operative patient group (*n* = 14), which may limit the generalizability of our findings. However, the wide confidence intervals observed in the pre-operative group quantitatively confirm the high uncertainty and variability in this population, whereas the narrower, non-zero intervals in the older adult group support the reliability of those findings despite the sample size. Nevertheless, a larger sample would be beneficial to better characterize the heterogeneity of responses in pathological gait populations.

Fourth, we did not investigate which type of participants’ data contribute most to improving model performance when included in the training set. Understanding the characteristics of informative participants would enable more efficient data collection strategies. Informative participants are those whose gait patterns, when included in training data, improve generalization to other individuals. Future studies should identify predictors of individual-level benefit from cross-population training, as well as characteristics of participants whose data enhances overall model generalization. This knowledge would guide clinical application by determining who should use cross-population versus individual-specific models, and would inform data collection strategies by identifying which participants to prioritize for training diverse, generalizable models.

Fifth, our results were obtained using a specific IMU device and configuration. Since IMU characteristics and embedded processing can differ across hardware, additional validation on other devices is needed before broad clinical deployment.

## 5. Conclusions

This study showed that the effectiveness of cross-population training for IMU-based joint angle estimation varies substantially across different populations and individuals. While cross-population training provided consistent benefits for older adults with statistically significant group-level improvement, it showed minimal impact on young adults who already achieved high baseline performance, and highly variable results in pre-operative patients. These findings indicate that the success of cross-population training depends critically on within-population gait heterogeneity: populations with diverse gait patterns benefit more from exposure to additional movement variability, while homogeneous populations see limited gains. For clinical application, our results suggest that cross-population models could be applied to older adults, individual-specific models may be sufficient for young healthy individuals, and pre-operative patients may require individual validation before deployment. However, these interpretations should be made cautiously given the limited sample size and controlled laboratory environment. Overall, this work provides preliminary insights for developing practical, generalizable gait analysis systems using minimal sensor configurations across diverse clinical populations.

## Figures and Tables

**Table 1:** Participant demographics.

Group	*n*	Age (Years)	Sex (M/F)
Young adults	17	21.8 ± 3.1 (20–28)	6/11
Older adults	20	73.3 ± 6.5 (65–88)	8/12
Pre-operative patients	14	75.7 ± 5.9 (67–88)	4/10

**Table 2:** Network architecture. The model consists of a convolutional stem, four residual stages, and a regression head. BN: batch normalization; GELU: Gaussian error linear unit; Ch.: channels.

Layer Type	Ch.	Kernel/Stride	Output
Input			
Input	6	–	6×200
Stem			
Conv1D → BN → GELU	64	7/1	64×200
MaxPool1D	64	3/2	64×100
Residual stages (BasicBlock)			
Stage 1: BasicBlock ×2	64	3/1	64×100
Stage 2: BasicBlock $\times {2\;}^{†}$	128	3/2	128×50
Stage 3: BasicBlock $\times {2\;}^{†}$	256	3/2	256×25
Stage 4: BasicBlock $\times {2\;}^{†}$	512	3/2	512×13
Regression Head			
GlobalAvgPool1D	512	–	512
Linear → GELU → Dropout (p=0.2) ×3	–	–	256→128→64
Linear (Output)	–	–	6

^†^ The first block in these stages uses stride 2 for downsampling, whereas the subsequent block uses stride 1.

**Table 3:** Joint-specific mean absolute error (MAE) comparison.

Group	Hip			Knee			Ankle			Average		
	Within	Cross	Δ	Within	Cross	Δ	Within	Cross	Δ	Within	Cross	Δ
Young (*n* = 17)	5.34	4.92	−0.42	4.54	4.40	−0.14	3.41	3.31	−0.10	4.43	4.21	−0.22
Older (*n* = 20)	3.97	3.78	−0.19	5.50	5.03	−0.47	3.41	3.32	−0.09	4.29	4.04	−0.25
Pre-operative (*n* = 14)	5.04	4.83	−0.21	6.25	6.02	−0.23	3.96	3.89	−0.07	5.08	4.91	−0.17

MAE in degrees, averaged across bilateral joints. Within = Within-population training, Cross = Cross-population training. Δ = Cross − Within. Negative values indicate improvement.

**Table 4:** Joint-specific Pearson correlation coefficient (R) comparison.

Group	Hip			Knee			Ankle			Average		
	Within	Cross	Δ	Within	Cross	Δ	Within	Cross	Δ	Within	Cross	Δ
Young (*n* = 17)	0.969	0.976	+0.007	0.970	0.973	+0.003	0.938	0.940	+0.002	0.959	0.963	+0.004
Older (*n* = 20)	0.961	0.969	+0.008	0.942	0.955	+0.013	0.894	0.902	+0.008	0.932	0.942	+0.010
Pre-operative (*n* = 14)	0.916	0.892	−0.024	0.911	0.896	−0.015	0.820	0.827	+0.007	0.882	0.872	−0.011

R values averaged across bilateral joints. Within = Within-population training, Cross = Cross-population training. Δ = Cross − Within. Positive values indicate improvement.

**Table 5:** Statistical comparison between cross-population and within-population training (MAE). ΔMAE = Cross − Within (negative indicates improvement).

Group	Mean ΔMAE (°)	95% CI	*p* (Wilcoxon)	*p* (Bonf.)	Effect Size
Young adults (*n* = 17)	−0.22±0.57	[−0.51,+0.02]	0.177	1.000	−0.33
Older adults (*n* = 20)	−0.25±0.33	[−0.39,−0.11]	**0.005**	**0.031**	−0.63
Pre-operative (*n* = 14)	−0.17±0.71	[−0.53,+0.19]	0.397	1.000	−0.23

Bold indicates statistical significance (p<0.05).

**Table 6:** Statistical comparison between cross-population and within-population training (Pearson correlation coefficient). ΔR = Cross − Within (positive indicates improvement).

Group	Mean ΔR	95% CI	*p* (Wilcoxon)	*p* (Bonf.)	Effect Size
Young adults (*n* = 17)	+0.004±0.006	[+0.001,+0.007]	**0.031**	0.188	+0.52
Older adults (*n* = 20)	+0.010±0.015	[+0.004,+0.017]	**0.002**	**0.013**	+0.69
Pre-operative (*n* = 14)	−0.011±0.063	[−0.046,+0.016]	0.778	1.000	+0.08

Bold indicates statistical significance (p<0.05).

**Table 7:** Cross-Validation Results: Within-population vs. Cross-population. Representative participants: Best (largest improvement in ΔMAE), Median (closest to median within-population MAE), and Worst (largest degradation in ΔMAE) for each group. Complete results for all participants are provided in Appendix B.

Participant ID	Within-Population		Cross-Population		Δ(Cross − Within)	
	MAE (↓)	R (↑)	MAE (↓)	R (↑)	ΔMAE	ΔR
Young adults (*n* = 17)						
Y10 (Best)	5.87	0.931	3.93	0.954	**−1.94**	**+0.023**
Y05 (Median)	4.46	0.962	4.11	0.968	**−0.35**	**+0.006**
Y12 (Worst)	3.60	0.956	4.24	0.956	+0.64	0.000
Older adults (*n* = 20)						
O08 (Best)	6.22	0.824	5.32	0.881	**−0.90**	**+0.058**
O09 (Median)	3.97	0.947	3.85	0.955	**−0.12**	**+0.009**
O11 (Worst)	3.39	0.960	3.70	0.946	+0.30	-0.014
Pre-operative (*n* = 14)						
P11 (Best)	8.03	0.795	6.48	0.857	**−1.55**	**+0.062**
P04 (Median)	4.58	0.919	4.48	0.939	**−0.10**	**+0.020**
P12 (Worst)	6.25	0.821	7.31	0.628	+1.06	−0.193

Bold values indicate improvement with cross-population training. MAE (↓): lower is better; R (↑): higher is better.

## Data Availability

The data presented in this study are available on request from the corresponding author due to privacy concerns.

## Appendix A. Participant Assignment to Cross-Validation Folds

Table A1 shows the detailed assignment of participants to the five cross-validation folds for each population group. Participants were randomly assigned to folds, and this assignment was fixed to ensure reproducibility across all experiments.

**Table A1:** Fixed random assignment of participants to five-fold cross-validation sets for each population group.

Fold	Young (*n* = 17)	Older (*n* = 20)	Pre-Operative (*n* = 14)
1	Y17, Y09, Y01, Y04	O20, O14, O18, O17	P09, P13, P03
2	Y06, Y08, Y05, Y14	O06, O16, O11, O08	P10, P04, P12
3	Y10, Y11, Y16	O15, O19, O02, O09	P11, P02, P05
4	Y15, Y02, Y07	O05, O07, O12, O01	P14, P06, P01
5	Y12, Y13, Y03	O10, O13, O03, O04	P07, P08
Total	17	20	14

## Appendix B. Complete Cross-Validation Results for All Participants

Table A2 presents the complete cross-validation results for all participants across both within-population and cross-population training strategies.

**Table A2:** Within- and cross-population cross-validation results for all participants. (Y: young adults, *n* = 17; O: older adults, *n* = 20; P: pre-operative patients, *n* = 14).

Participant ID	Within-Population		Cross-Population		Δ(Cross − Within)	
	MAE (↓)	R (↑)	MAE (↓)	R (↑)	ΔMAE	ΔR
Y01	6.60	0.930	6.70	0.931	+0.10	**+0.001**
Y02	3.77	0.945	3.36	0.954	**−0.41**	**+0.009**
Y03	2.91	0.973	2.99	0.973	+0.08	0.000
Y04	3.77	0.956	3.88	0.958	+0.11	**+0.002**
Y05	4.46	0.962	4.11	0.968	**−0.35**	**+0.006**
Y06	5.99	0.965	6.19	0.965	+0.19	0.000
Y07	3.03	0.970	3.20	0.970	+0.17	0.000
Y08	4.80	0.961	4.71	0.961	**−0.09**	0.000
Y09	5.06	0.931	4.83	0.941	**−0.23**	**+0.009**
Y10	5.87	0.931	3.93	0.954	**−1.94**	**+0.023**
Y11	4.28	0.973	4.11	0.973	**−0.17**	0.000
Y12	3.60	0.956	4.24	0.956	+0.64	0.000
Y13	4.73	0.966	4.86	0.960	+0.13	−0.006
Y14	3.59	0.970	3.53	0.970	**−0.05**	0.000
Y15	5.18	0.970	4.21	0.970	**−0.97**	0.000
Y16	4.71	0.969	4.09	0.975	**−0.62**	**+0.006**
Y17	2.90	0.973	2.59	0.977	**−0.30**	**+0.004**
O01	3.32	0.960	3.30	0.961	**−0.02**	**+0.002**
O02	3.27	0.955	2.96	0.957	**−0.31**	**+0.001**
O03	4.26	0.950	3.59	0.959	**−0.67**	**+0.010**
O04	3.64	0.958	3.56	0.963	**−0.08**	**+0.004**
O05	2.70	0.969	2.69	0.974	**−0.01**	**+0.005**
O06	4.67	0.956	4.38	0.965	**−0.29**	**+0.008**
O07	3.61	0.939	3.86	0.931	+0.24	−0.008
O08	6.22	0.824	5.32	0.881	**−0.90**	**+0.058**
O09	3.97	0.947	3.85	0.955	**−0.12**	**+0.009**
O10	2.74	0.962	2.78	0.967	+0.04	**+0.005**
O11	3.39	0.960	3.70	0.946	+0.30	−0.014
O12	8.86	0.785	8.36	0.827	**−0.50**	**+0.042**
O13	3.66	0.956	3.65	0.960	**−0.01**	**+0.004**
O14	3.98	0.950	3.43	0.957	**−0.55**	**+0.007**
O15	4.54	0.931	3.91	0.941	**−0.63**	**+0.009**
O16	5.71	0.926	5.77	0.938	+0.06	**+0.012**
O17	3.84	0.931	3.09	0.946	**−0.74**	**+0.014**
O18	4.33	0.890	4.23	0.913	**−0.10**	**+0.024**
O19	5.94	0.899	5.69	0.901	**−0.24**	**+0.002**
O20	3.13	0.972	2.71	0.974	**−0.42**	**+0.003**
P01	4.74	0.893	3.90	0.916	**−0.84**	**+0.023**
P02	4.89	0.936	4.28	0.949	**−0.61**	**+0.013**
P03	5.23	0.903	4.16	0.898	**−1.06**	−0.005
P04	4.58	0.919	4.48	0.939	**−0.10**	**+0.020**
P05	4.04	0.927	4.38	0.928	+0.34	**+0.001**
P06	4.71	0.878	4.83	0.888	+0.12	**+0.010**
P07	5.13	0.746	5.48	0.710	+0.35	−0.036
P08	4.82	0.830	5.39	0.756	+0.57	−0.074
P09	4.15	0.961	3.71	0.967	**−0.44**	**+0.006**
P10	4.58	0.912	4.00	0.931	**−0.58**	**+0.019**
P11	8.03	0.795	6.48	0.857	**−1.55**	**+0.062**
P12	6.25	0.821	7.31	0.628	+1.06	−0.193
P13	6.60	0.901	6.96	0.888	+0.36	−0.013
P14	3.35	0.946	3.40	0.944	+0.05	−0.002

Bold values indicate improvement with cross-population training. MAE (↓): lower is better; R (↑): higher is better.
